# Determination of Inorganic Cations and Anions in Chitooligosaccharides by Ion Chromatography with Conductivity Detection

**DOI:** 10.3390/md15020051

**Published:** 2017-02-22

**Authors:** Lidong Cao, Xiuhuan Li, Li Fan, Li Zheng, Miaomiao Wu, Shanxue Zhang, Qiliang Huang

**Affiliations:** 1 Institute of Plant Protection, Chinese Academy of Agricultural Sciences, No. 2 Yuanmingyuan West Road, Beijing 100193, China; caolidong@caas.cn (L.C.); lixiuhuan0822@163.com (X.L.); zhengli7seven@163.com (L.Z.); wumiaomiao2016@163.com (M.W.); 2 Institute of Quality Standards & Testing Technology for Agro-Products, Chinese Academy of Agricultural Sciences, No. 12 Zhongguancun South Street, Beijing 100081, China; leefan66@126.com; 3 Hainan Zhengye Zhongnong High-Tech Co., LTD., No. 25 Nansha Road, Haikou 570206, Hainan, China; zhangshanxue@zyzn.net

**Keywords:** chitooligosaccharides, ion chromatography, inorganic cations and anions, method validation, quantification

## Abstract

Chitooligosaccharides (COSs) are a promising drug candidate and food ingredient because they are innately biocompatible, non-toxic, and non-allergenic to living tissues. Therefore, the impurities in COSs must be clearly elucidated and precisely determined. As for COSs, most analytical methods focus on the determination of the average degrees of polymerization (DPs) and deacetylation (DD), as well as separation and analysis of the single COSs with different DPs. However, little is known about the concentrations of inorganic cations and anions in COSs. In the present study, an efficient and sensitive ion chromatography coupled with conductivity detection (IC-CD) for the determination of inorganic cations Na^+^, NH_4_^+^, K^+^, Mg^2+^, Ca^2+^, and chloride, acetate and lactate anions was developed. Detection limits were 0.01–0.05 μM for cations and 0.5–0.6 μM for anions. The linear range was 0.001–0.8 mM. The optimized analysis was carried out on IonPac CS12A and IonPac AS12A analytical column for cations and anions, respectively, using isocratic elution with 20 mM methanesulfonic acid and 4 mM sodium hydroxide aqueous solution as the mobile phase at a 1.0 mL/min flow rate. Quality parameters, including precision and accuracy, were fully validated and found to be satisfactory. The fully validated IC-CD method was readily applied for the quantification of various cations and anions in commercial COS technical concentrate.

## 1. Introduction

Chitooligosaccharides (COSs) derive from the hydrolysis of chitosan, a cationic polysaccharide obtained by partial deacetylation of chitin, the second most abundant naturally occurring homopolysaccharide extracted from, among others, the exoskeleton of crustaceans and insects and fungal cell walls [1]. COSs are readily soluble in water due to their shorter chain lengths and free amino groups in d-glucosamine units. The greater solubility and low viscosity of COSs at neutral pH make COSs perform remarkable biological activities at the cellular or molecular level [2]. Moreover, COSs are promising as a drug candidate, and as a food ingredient, additive, and preservative that improve food quality and human health, because they are innately biocompatible, non-toxic, and non-allergenic to living tissues [3,4,5].

As a potential candidate for practical application in medicine and food, oral administration of COSs is inevitable. It has been claimed that COSs reach systemic circulation after oral administration [6]. Therefore, for the sake of human health, impurities in COSs, especially inorganic ions, must be clearly elucidated and precisely determined. COSs are produced by the enzymatic or alkaline heating deacetylation of chitosan or chitin [7]. The inherently present inorganic cations, such as sodium (Na^+^), potassium (K^+^), calcium (Ca^2+^), magnesium (Mg^2+^), and ammonium (NH_4_^+^) in raw material possibly persist in the final product of COSs. The free amino groups in COSs can undergo aerobic oxidation due to the lone electron pair of nitrogen atoms, leading to the decomposition of COSs during storage. This issue could be addressed by changing the neutral amino group to ammonium with the assistance of inorganic or organic acid during the manufacturing process. Hydrochloric, acetic, and lactic acid are the most commonly used acids. Thus, the counter anions of COSs should be qualitatively and quantitatively determined.

It is clear that mineral elements, such as Na^+^, K^+^, Ca^2+^, and Mg^2+^, are essential nutrients that function in the regulation of cardiac output and peripheral vascular resistance, which are the main determinants of blood pressure level [8]. There is a balanced concentration level for all these elements in order to maintain the essential functions of the human body [9,10]. Deficiency or excess of these elements over the required level can have implications for human health. As COSs are a promising candidate for the food and medicine fields, their inorganic cations and anions should be strictly monitored and controlled, which could improve the beneficial effects and avoid deleterious effects. Consequently, the development of an efficient method for the determination of inorganic cations and anions in COS samples is important from nutritional, toxicological, and technological points of view.

A variety of methods have been developed for analyzing such mineral inorganic cations in different matrices, including popularly used atomic spectroscopic methods such as atomic absorption spectroscopy (AAS) [11,12], inductively coupled plasma–mass spectrometry (ICP-MS) [13], atomic emission spectrometry (ICP-AES) [14], and optical emission spectrometry (ICP-OES) [15], as well as capillary electrophoresis (CE) [16]. However, some of these methods suffer from spectral and chemical interferences, asynchronous determination of mixed cations, laborious and prolonged procedures for sample preparation, as well as utilization of toxic concentrated acid and other reagents. Capillary electrophoresis has limits of poor reproducibility of migration times and peak areas, and moderate sensitivity [17]. Due to the sensitivity, stability of the separation system, good selectivity, and capacity of multi-element analysis in a single run, ion chromatography coupled with conductivity detection (IC-CD) has become the method of choice for the separation and determination of multiple cations and anions in various sample matrices [18,19,20]. This powerful and reliable technique has been widely used for analysis of Na^+^, K^+^, Ca^2+^, Mg^2+^, and anions in biological samples [21], food [22,23], biodiesel [24,25,26], oil [27], plant extract [28], and water [29,30,31], particularly at the level of trace concentrations.

As for COSs, most of the analytical methods focus on the determination of the average degrees of polymerization (DPs) and deacetylation (DD), as well as separation and analysis of the single COSs with different DPs [7]. Recently, we reported an efficient and sensitive analytical method based on high performance anion exchange chromatography with pulsed amperometric detection (HPAEC-PAD) for the simultaneous separation and determination of glucosamine and COSs, with DPs ranging from 2 to 6 without prior derivatization [32]. However, little is known about the concentrations of Na^+^, NH_4_^+^, K^+^, Mg^2+^, Ca^2+^, and anions in COSs. In the present study, an IC-CD method for determination of inorganic cations Na^+^, NH_4_^+^, K^+^, Mg^2+^, Ca^2+^, and chloride, acetate and lactate anions was developed. The detection is performed using conductimetry. Parameters such as linearity, sensitivity, precision, and accuracy were fully validated. Moreover, this validated method was used to quantify the inorganic cations and anions in COS technical concentrate. The procedure is simple and environmentally friendly because only water is used to dissolve the COS samples.

## 2. Results and Discussion

### 2.1. Optimization of Chromatographic Conditions

**Figure 1:**
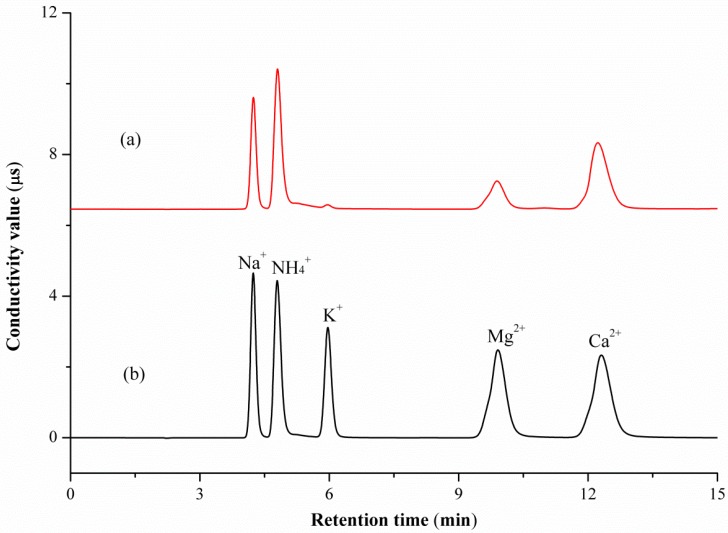
Representative ion chromatography with conductivity detection (IC-CD) chromatograms of cations in COS technical concentrate sample (**a**) and a mixed standard solution (**b**).

**Figure 2:**
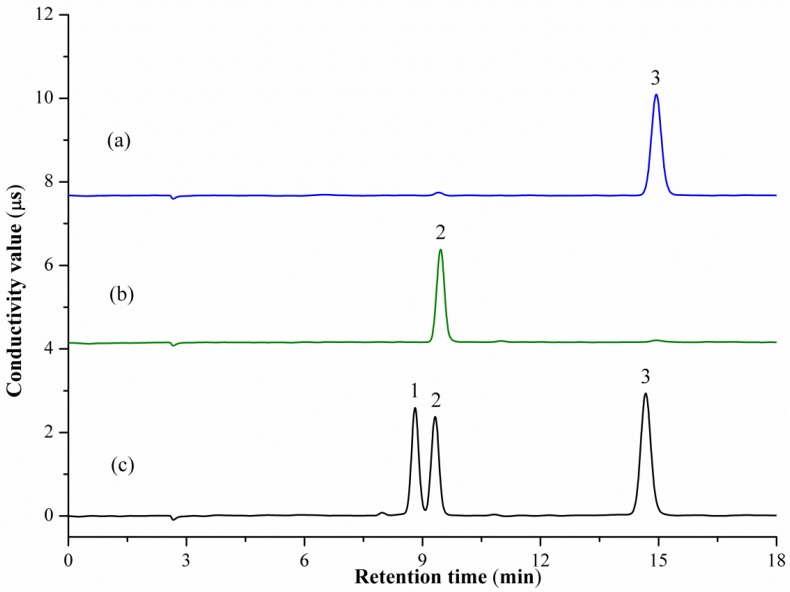
Representative IC-CD chromatograms of anions in COS technical concentrate sample (**a**), sample (**b**), and a mixed standard solution (**c**). Peaks: (1) lactate; (2) acetate; (3) chloride.

Ion chromatography is the most popular analytical method used for the determination of anions and cations in various sample matrices. Satisfactory separation depends mainly on the column, mobile phase, and flow rate. These three variables were screened during optimization of chromatographic conditions, which was carried out using mixed cations or anions standard solutions. The IonPac CS12A and IonPac AS12A analytical columns were used for cations and anions separation, respectively. The flow rate was set to 1.0 mL/min for both cations and anions optimization. The results demonstrate that the isocratic elution with 20 mM methanesulfonic acid solution enabled a satisfactory separation for Na^+^, NH_4_^+^, K^+^, Mg^2+^, and Ca^2+^ within 15 min. While peak tailing and longer retention time occurred when 15 mM mobile phase was used. Isocratic elutions with 4, 7, 10, 15, and 20 mM sodium hydroxide solution were employed for anions separation. The results indicated that a 4 mM mobile phase can improve resolution for chloride, acetate, and lactate anions with 15 min. The resolutions for acetate and lactate were not satisfactory when isocratic elution with other concentrations were used. Representative IC-CD chromatograms of the mixed cations and anions standard are shown in Figure 1b and Figure 2c, where the signals of Na^+^, NH_4_^+^, K^+^, Mg^2+^, Ca^2+^, and chloride, acetate, and lactate anions are clearly shown.

### 2.2. Calibration and Method Validation

Quality parameters such as sensitivity, linearity, precision, and accuracy were fully evaluated. Results showed that the linear ranges for cations and anions were 0.002–0.8 mM and 0.001–0.6 mM, respectively. All the calibration curves showed good linearity (*R*^2^ = 0.9950–0.9999) in the tested range (Table 1). The limits of detection (LOD) and quantification (LOQ) were defined as the minimum amounts at which the analyte can be reliably detected and quantified. Typical signal-to-noise (S/N) ratios of the LOD and LOQ were 3 and 10, respectively. Diluted low concentrations of the cations and anions standard solutions were injected to determine the S/N ratio. Then, the LOD and LOQ were calculated. For cations, the LOD and LOQ ranged from 0.01 to 0.05 (corresponding to 0.25–1.25 pmol) and 0.03 to 0.15 μM (corresponding to 0.75–3.75 pmol), respectively. For anions, the LOD and LOQ were 0.5 μM (corresponding to 12.5 pmol) and 1.6 μM (corresponding to 40 pmol), respectively.

Precision was evaluated by measuring known amounts of the mixed cations and anions standards and the real sample determination. To establish repeatability (intraday) and intermediate (interday) precision, variations in terms of peak areas and retention times of the mixed standard solutions at three concentration levels were determined (Table 2). Repeatability was assessed using seven replicates in one day. Under repeatability conditions, retention times and integrated peak areas of all tested analytes were stable with 0.1–1.6 and 0.2–3.0 %RSD, respectively. Intermediate precision was assessed from nine determinations (three determinations daily over three days) using the same equipment, but performed on three consecutive days using three separately prepared batches of eluents. Under intermediate precision conditions, retention times and integrated peak areas of all tested analytes were stable with 0.3–3.7 and 0.2–6.2 %RSD, respectively. These values are slightly higher than what was found for repeatability. Method precision was also assessed by comparing the variations among seven replicates determinations of the same batch of COS technical concentrate with the Horwitz value (%RSDr). All the %RSD values of cations and anions determinations were less than the corresponding %RSDr (Table 3), indicating that the developed method is precise.

The accuracy was evaluated through the standard addition method under optimized conditions, and it was found to be satisfactory with the recoveries ranging from 86.0% to 110.7% under three spiked concentration levels (Table 4). These validation results indicate that this IC-CD method is sensitive, precise, and accurate for the simultaneous quantitative determination of Na^+^, NH_4_^+^, K^+^, Mg^2+^, Ca^2+^, or chloride, acetate and lactate anions.

### 2.3. Analysis of Cations and Anions in COS Technical Concentrates

During the manufacture of COS technical concentrate, the existing inorganic cations, such as Na^+^, NH_4_^+^, K^+^, Mg^2+^, and Ca^2+^ in the raw-material or incomplete desalination process, may result in the inorganic cations impurities in the final product of COSs. For longer shelf life, commercial COSs are usually present in its ammonium salt form. Hydrochloric, acetic, and lactic acids are the most commonly used acids. Figure 1a and Figure 2a,b show ion chromatograms obtained in the analysis of two COS technical concentrates where the signals of existing cations and anions are clearly indicated. Moreover, as presented in Table 3, all the inorganic cations of Na^+^, NH_4_^+^, K^+^, Mg^2+^, and Ca^2+^ were detected in COS technical concentrate. Among the detected cations, NH_4_^+^ is the cation that has the highest concentration in both COS samples, followed by Ca^2+^. The content of K^+^ is the lowest. For anions, chloride and acetate were detected in COS Technical Concentrates A and B, with concentrations of 17.64% and 11.57%, respectively. As potential food and medicine field candidates, the ammonium lactate of COSs will be an ideal combination due to its biocompatibility, its low-toxicity, and the biodegradability of the lactic acid. Although the determination of lactate anion for a real COS sample was not performed, the recovery test clearly indicated that the proposed method is applicable for lactate anion analysis in COS samples.

## 3. Materials and Methods

### 3.1. Materials

Sodium hydroxide solution (50%, *w*/*w*) was purchased from Alfa Aesar Co., Ltd. (Tianjin, China). Methanesulfonic acid (99%) and sodium acetate (99%) were obtained from Sigma Aldrich Co. LLC. (Shanghai, China). Potassium chloride (99%), ammonium chloride (99%), magnesium sulfate (99%), calcium chloride (96%), and lactic acid (98%) were purchased from Sinopharm Chemical Reagent Beijing Co., Ltd. (Beijing, China). All stock standard solutions of anions and cations (20 mM) were prepared directly from the analytical reagent grade chemicals (as purchased) using deionized water, which was obtained using a MilliQ (Millipore, Bedford, MA, USA) water purification system. The working standard solutions were prepared as needed by appropriately diluting concentrated stock solutions with water. COS Technical Concentrate A with a number-average molecular weight (Mn) of 868 and a degree of deacetylation (DD) of 95% was provided by Hainan Zhengye Zhongnong High-Tech Co., Ltd. (Haikou, China). COS Technical Concentrate B with an Mn of 673 and a DD of 92% was obtained from Qingdao Zhongda Agricultural Science and Technology Co. Ltd. (Qingdao, China). The Mn was determined by matrix-assisted laser desorption/ionization time-of-flight mass spectrometry (MALDI-TOF-MS) analysis. The DD was determined by acid-base titration with bromocresol green as indicator. COS technical concentrate was accurately weighed and dissolved in water to prepare stock sample solution. The working sample solutions were prepared by dilution with water. All standards and samples solutions were stored in polyethylene bottles.

### 3.2. Chromatographic Analysis

All chromatographic analysis were performed using a Dionex ICS-3000 (Sunnyvale, CA, USA) system composed of an AS40 automated sampler, a GP40 gradient pump, and a CD20 conductivity detector. In order to reduce the background eluent conductivity, the detector was preceded by a Dionex self regenerating suppressor system. Suppression was achieved with a Dionex ASRS-300 (4 mm) for the anions and CSRS-300 (4 mm) for the cations. The ion separation was carried out with two different ion-exchange columns. Anions were separated on an IonPac AS12A column (250 mm × 4 mm i.d.) protected by an IonPac AG12A guard column (50 mm × 4 mm i.d.). Cations were determined using an IonPac CS12A column (250 mm × 4 mm i.d.) equipped with an IonPac CG12A guard column (50 mm × 4 mm i.d.). Data were collected using a Chromeleon 6.8 chromatogram workstation.

All eluents were degassed and pressurized under high-purity nitrogen to prevent dissolution of carbon dioxide and subsequent production of carbonate. An aqueous solution containing 20 mM methanesulfonic acid was used for elution of cations. An aqueous solution containing 4 mM sodium hydroxide served as eluent for anions. Elution was carried out at a flow rate of 1.0 mL/min and 25 μL was injected for both anion and cation determinations. The concentrations of each cations and anions in the samples were calculated using a calibration curve that produced the relationship between the amount of analyte and the peak area. All analyses were carried out in duplicate.

### 3.3. Calibration

To assess the linearity, calibration curves were plotted by a partial least squares method on the analytical data of peak area and concentration, using analyte standards covering the concentration range of 0.002–0.8 mM for Na^+^, NH_4_^+^, K^+^, Mg^2+^, and Ca^2+^, and 0.001–0.6 mM for chloride, acetate and lactate anions. The linear range of the curve was assessed by the value of linear correlation coefficient and the residuals. The dilute standard solution was further diluted to known low concentration with water for signal-to-noise (S/N) ratio determination. The limits of detection (LOD) and quantification (LOQ) were defined as the minimum concentrations resulting in signal-to-noise (S/N) ratios of 3 and 10, respectively.

### 3.4. Method Validation

The method precision was evaluated according to the repeatability (intraday) and intermediate (interday) precision and was expressed as a relative standard deviation (%RSD). For the mixed cations or anions standard solution, the precision in terms of retention time and peak area was determined. Repeatability was assessed using seven replicates in one HPLC run. Intermediate precision was evaluated from nine determinations (three determinations daily over three days) using the same equipment, but performed on three consecutive days using three separately prepared batches of mobile phase. Both repeatability and intermediate precision were determined at three concentrations levels for cations (0.04, 0.08, and 0.12 mM) and anions (0.13, 0.19, and 0.25 mM). In addition to the standard solutions, the precision was also evaluated by the real sample determination. For the COS technical concentrate sample, the contents of the containing cations and anions were measured under the prescribed conditions. The coefficients of variations of seven replicate determinations of the same batch of COS technical concentrate are compared with the Horwitz value (%RSDr) [33]. The Horwitz equations are described as follows:

(1) $${\%\mathrm{RSD}}_{R}=2^{\left(1-0.5\log_{10}C\right)}$$

(2) $$\%\mathrm{RSDr}={\%\mathrm{RSD}}_{R}\times 0.67$$

where %RSD_R_ represents the inter-laboratory coefficient of variation (CV), %RSDr represents the repeatability CV, and C represents the concentration of the analyte in the sample as a decimal fraction.

The method accuracy was determined by spike-recovery test. A known amount of the cation or anion working standard solution was added to a predetermined amount of the COS technical concentrate, and the spiked sample was assayed. The total amount of each analyte was calculated from the corresponding calibration curve, and recovery was calculated using the following formula: recovery (%) = (observed amount − original amount)/spiked amount × 100%. The samples of COS technical concentrate were spiked with the analytes at three different concentrations. Three determinations were performed for each standard addition. Each determination was injected in duplicate.

### 3.5. Method Application

To determine the cations and anions, COS technical concentrate was accurately weighed and dissolved in water to prepare stock sample solution. The working sample solutions were prepared by dilution with water. For sample preparation, seven replicates were performed. For cations and anions determination, the concentration of the working sample solution was approximately 2000 and 50 mg/L, respectively.

## 4. Conclusions

The increasing interest of COSs in the food and medicine fields implies a need to control the quality of the product so that undesirable health effects are avoided. In the present work, an efficient, sensitive, and quick IC-CD method was established and demonstrated as suitable for separating, identifying, and quantifying inorganic cations of Na^+^, NH_4_^+^, K^+^, Mg^2+^, Ca^2+^, and chloride, acetate and lactate anions within 15 min. High sensitivity, satisfactory linearity, precision, and accuracy were achieved. The proposed method was readily applied for quantitative determination of the cations and anions stated above, providing a very useful method for the analysis of COSs for quality control and biological research purposes.

## Figures and Tables

**Table 1:** Calibration parameters for cations and anions in standard solutions.

Analyte		Linear Range (mM)	Calibration Curve ^a^	*R*^2^	LOD (μM )	LOQ (μM)
Cation	Sodium	0.002–0.8	*y* = 6.4217*x* + 0.0073	0.9999	0.01	0.03
	Ammonium ^b^	0.002–0.1	*y* = 5.2160*x* + 0.0176	0.9960	0.02	0.06
	Potassium	0.002–0.8	*y* = 7.6307*x* − 0.0009	0.9998	0.04	0.14
	Magnesium	0.002–0.8	*y* = 13.3560*x* + 0.0130	0.9999	0.05	0.15
	Calcium	0.002–0.8	*y* = 14.4640*x* + 0.0256	0.9999	0.02	0.05
Anion	Lactate	0.001–0.6	*y* = 5.3829*x* − 0.0317	0.9998	0.6	2.0
	Acetate	0.001–0.6	*y *= 3.9231*x* + 0.0674	0.9950	0.5	1.7
	Chloride	0.001–0.6	*y* = 9.1727*x* − 0.1074	0.9988	0.5	1.6

^a^ *y* and *x* refer to the signal response (μS) and molar concentration (mM), respectively. ^b^ The calibration curve is *y* = 2.9823*x* + 0.2796 at the linear range 0.13–0.5 mM and the *R*^2^ is 0.9961. LOD: limit of detection; LOQ: limit of quantification.

**Table 2:** Determination of method precision under repeatability (intraday) and intermediate precision (interday) conditions given as RSD(%) of peak area and retention time.

Analyte		Repeatability (*n* = 7)						Intermediate Precision (*n* = 9)					
		Peak Area			Retention Time			Peak Area			Retention Time		
		C1	C2	C3	C1	C2	C3	C1	C2	C3	C1	C2	C3
Cation	Sodium	0.39	0.21	0.60	0.46	0.07	0.61	0.94	6.44	4.57	0.49	0.39	0.40
	Ammonium	0.49	0.44	0.23	0.12	0.06	0.66	0.57	0.51	0.18	0.44	0.32	0.65
	Potassium	0.87	0.51	0.28	0.44	0.06	0.12	0.81	0.54	0.26	0.50	0.33	0.34
	Magnesium	1.13	0.70	0.19	0.67	0.13	0.21	1.39	0.82	0.18	1.14	1.30	0.64
	Calcium	2.79	0.75	0.37	0.63	0.08	0.17	3.08	1.20	0.47	1.44	0.79	0.63
Anion	Lactate	0.85	0.48	3.04	1.03	0.62	1.52	6.20	4.69	5.60	0.84	0.68	1.64
	Acetate	0.64	0.48	1.46	1.14	0.62	1.56	0.84	0.56	2.39	3.69	0.56	1.37
	Chloride	0.52	0.62	0.83	1.18	0.59	0.63	5.13	4.43	6.68	1.00	0.62	1.65

RSD: Relative standard deviation. For cations: C1 (mM): 0.04; C2 (mM): 0.08; C3 (mM): 0.12. For anions: C1 (mM): 0.13; C2 (mM): 0.19; C3 (mM): 0.25.

**Table 3:** Determination of each cation and anion in COS technical concentrates.

Analyte		COS Technical Concentrate A			COS Technical Concentrate B		
		Content (%) ^a^	%RSD	%RSDr	Content (%) ^a^	%RSD	*%*RSDr
Cation	Sodium	0.08	2.13	3.91	0.08	2.33	3.91
	Ammonium	0.39	1.18	3.08	0.36	2.71	3.14
	Potassium	0.01	4.91	5.24	0.01	4.95	5.24
	Magnesium	0.04	2.72	4.37	0.04	3.35	4.37
	Calcium	0.17	3.04	3.50	0.18	2.86	3.48
Anion	Acetate	17.64	1.30	1.74	–	–	–
	Chloride	–	–	–	11.57	0.34	1.85

^a^ Mass percentage of each cation and anion in COS technical concentrate (means value of seven determinations).

**Table 4:** Method accuracy for determining cations and anions in COS technical concentrates.

Analyte		Recovery (%)		
		Spiked C1	Spiked C2	Spiked C3
Cation	Sodium	102.13 ± 4.47	103.29 ± 3.45	98.79 ± 1.41
	Ammonium	89.68 ± 4.64	90.21 ± 2.13	87.80 ± 0.41
	Potassium	86.41 ± 0.37	90.51 ± 0.22	92.54 ± 1.82
	Magnesium	93.08 ± 2.19	97.02 ± 1.73	96.94 ± 3.02
	Calcium	91.90 ± 0.45	86.04 ± 0.24	93.42 ± 0.39
Anion	Lactate	110.65 ± 2.84	105.64 ± 4.79	107.14 ± 3.40
	Acetate	103.17 ± 3.04	108.97 ± 2.24	102.43 ± 6.03
	Chloride	97.97 ± 4.51	105.84 ± 3.87	108.15 ± 0.94

All values were given as mean recovery (*n* = 3) ± SD. SD: standard deviation. For cations: C1 (mM): 0.04; C2 (mM): 0.08; C3 (mM): 0.12. For anions: C1 (mM): 0.13; C2 (mM): 0.19; C3 (mM): 0.25.
